# A transatlantic cleavage in sport regulation: French policy responses to the Enhanced Games

**DOI:** 10.3389/fspor.2026.1898543

**Published:** 2026-09-14

**Authors:** Joseph Godefroy, Baptiste Viaud

**Affiliations:** 1 UMR CNRS 6025, Nantes, France; 2 Nantes Universite, Nantes, France

**Keywords:** Enhanced Games, governance, labor, olympism, sport, doping, human enhancement, moral economy

## Abstract

This article examines how the Enhanced Games (EG), a sporting competition explicitly permitting the use of performance-enhancing pharmacological substances, destabilize national sport regulation in France. Drawing on the participation of French sprinter Mouhamadou Fall, the only French athlete officially engaged in the inaugural edition of the EG, we analyze the rhetorical and institutional confrontations triggered by his public announcement of October 23, 2025. The public stance taken by an elite athlete already sanctioned for doping and unlicensed since 2023 compelled sporting authorities to make their moral and regulatory boundaries explicit. The study combines three empirical sources: Fall's public statements between October 2025 and May 2026 (press interviews, a long-form YouTube interview, an EG promotional video, and Instagram posts, *n* = 54); a review of the French press from June 2023 onward (*n* = 97); and institutional communications from French and international sporting and anti-doping bodies (*n* = 4). Results show that Mouhamadou Fall deploys three registers of legitimation: bodily autonomy, medical supervision, and fair remuneration for sporting labor. French institutions respond with a coordinated normative reaffirmation, without engaging substantively with the health or economic questions raised. This case reveals a displacement of the doping controversy toward a dispute over status, value, and the governance of sporting labor, and crystallizes a transatlantic cleavage between the European tradition of public regulation and a market-driven logic of performance contracting.

## 1 Introduction

In June 2023, the announcement of the Enhanced Games (EG), a sporting competition in which the use of performance-enhancing pharmacological substances is not merely tolerated but institutionally encouraged, was received with marked skepticism in the French press. Dismissed by some commentators as “a utopian project of dangerous entrepreneurs” and by World Athletics president Sebastian Coe as “a load of rubbish,” the event was initially presented as an epiphenomenon without consequence. Yet the inaugural edition, staged on May 26, 2026, at the Resorts World Hotel in Las Vegas, has since established itself in the international sporting calendar. Backed by a consortium of technology entrepreneurs (Peter Thiel, Christian Angermayer, and Balaji Srinivasan) and listed on the Nasdaq at a valuation of $1.2 billion, the Enhanced Games company exemplifies what Olivier Alexandre has described as “tech start-ups” that “set out to remake the world” (1).

The Enhanced Games do not merely propose an alternative competition format: they constitute an ideological challenge to the institutional order of modern sport (2). The International Olympic Committee (IOC), the World Anti-Doping Agency (WADA), and the international federations that have historically governed legitimate performance are directly attacked at their foundations. In their place, EG promoters advocate what they term “human enhancement,” a notion whose reference formulation was proposed by Savulescu and Bostrom (3): pharmacologically assisted optimization of physical capacities, presented simultaneously as a scientific frontier, a commercial opportunity, and a fundamental right. On May 22, 2025, WADA published a communiqué condemning the Enhanced Games as “dangerous and irresponsible,” while the IOC and WADA athlete commissions jointly denounced the event on June 10, 2025, as “contrary to sporting integrity”.

This article takes October 23, 2025, as its point of departure, the day French sprinter Mouhamadou Fall publicly announced his participation in the Enhanced Games as a salaried athlete. Disclosed first through his social media channels, then developed in an interview with *Le Monde*, this declaration constituted an unprecedented challenge to France's sporting institutions. The Ministry of Sports, Youth and Associative Life (MSJVA), the French National Olympic and Sports Committee (CNOSF), and the French Anti-Doping Agency (AFLD) responded on October 24 with a joint tripartite statement; the French Athletics Federation (FFA) issued a separate communiqué. The “Fall case” was perceived as a threat to sporting order, forcing previously silent institutions to make their normative boundaries explicit, rendering visible the power relations ordinarily implicit in sports governance.

Situated within a sociology of sporting labor (4, 5) and a moral economy of doping (6, 7), the article is organized around three questions: (1) What biographical and structural conditions made Mouhamadou Fall's participation in the EG possible? (2) How do French institutions position themselves in response, and what does this response reveal about the limits of their regulatory authority? (3) What registers of justification does Mouhamadou Fall deploy to legitimize his engagement?

The institutional “panic” provoked by the EG and by the announcement of French participation reveals, first, the considerable anxiety of sporting movement leaders. Having made their distance from money a symbol of Olympic values (8), their haste to denounce this spectacularization of performance attests, conversely, to the relative fragility of their position within a market economy that the EG open dangerously to competition. At the same time, the counter-offensive mounted by French sport leaders proves highly performative: it discredits this “masquerade” while reasserting their full authority. By framing the debate in terms of values and defending “sincere” sport (9), they cast engagement with this enterprise as morally condemnable conduct. In denouncing the venality of EG employees, they ultimately foreclose any discussion of the recognition and remuneration of sporting labor, the very issues raised by EG athletes, including Mouhamadou Fall.

## 2 Method

This study adopts a qualitative sociological approach, combining biographical analysis of a singular case with analysis of institutional discourse. The empirical corpus was built in three parts.

The first comprises Mouhamadou Fall's public statements between October 2025 and May 2026: an interview published in Le Monde on October 25, 2025; a long-form interview conducted by the YouTube channel Younivers; a promotional video produced by the EG entitled “$1,000,000 Sprint: Mouhamadou Fall's Race to Become the Fastest Human Alive”; and 54 Instagram posts. These materials were subjected to thematic discourse analysis, with particular attention to the argumentative frameworks deployed and their variation according to the context of production.

The second component comprises 97 French press articles published between June 2023 and May 2026, drawn from L’Équipe, Le Monde, Le Point, 20 Minutes, Ouest-France, and BFM Sport, among others. This corpus was assembled through a systematic search in the Europresse database using the entries “Enhanced Games,” “Mouhamadou Fall,” and “doping,” over the period from June 1, 2023, to May 31, 2026. Duplicates and verbatim wire-copy reproductions were discarded. This corpus enables reconstruction of the evolution of French media framing of the Enhanced Games phenomenon over time, and contextualizes the specific conjuncture triggered by Fall's announcement.

The third part of the materials comprises 4 institutional communications: the WADA communiqué of May 22, 2025; the joint statement of the IOC and WADA athlete commissions of June 10, 2025; the MSJVA/CNOSF/AFLD joint declaration of October 24, 2025; and the FFA communiqué of October 24, 2025, signed by president Jean Gracia. Analysis focused on the normative registers deployed, declared sanction threats, and the models of sporting practice defended.

## 3 Results

### 3.1 Mouhamadou Fall: an athlete fitted to the Enhanced Games

Mouhamadou Fall was born on February 25, 1992, in Beaumont-sur-Oise, in the Paris region. The second-to-last of twelve siblings, he retired from amateur football at 19 to manage a hookah bar with his brothers. He ran his first 100 m at 23, after being spotted in informal challenges among friends. His debut official 100-m time of 10.90 presaged nothing of the progression that followed: within five years, coached successively by Anthony Yaïch and then by Mickaël Hanany (his current trainer, based in El Paso, Texas), Fall became one of the best French sprinters of his generation: four-time French champion in the 100 m (2020, 2021, 2022, 2023) and two-time champion in the 200 m (2019 and 2021). This trajectory as a “late entrant” (tard venu) placed Mouhamadou Fall in a particular position vis-à-vis French athletics institutions. He had not come through the usual talent development pipelines, was unknown in athletics circles, and arrived among the elite without mastering the field's codes: running his first official sprint in football kit, his family cheering from the stands with drums, refusing to leave the track after a false start. This outsider status explains the rejection, in 2017, of his application to join the Institut National du Sport, de l’Expertise et de la Performance (INSEP). While the official justification cited “insufficient level,” Mouhamadou Fall, for his part, interprets this refusal as a sanction for his exteriority to the world of athletics. Our materials do not allow us to decide between these two readings. They do establish, however, that this refusal constitutes, in the narrative he offers today, the first term in a series of exclusions.

This rapid progression, achieved entirely outside the usual federal structures, nourished an early sense of institutional exclusion. In his interview with Younes Nezar (Younivers), Fall revisits episodes he retrospectively reconstructs as evidence of long-standing rejection: non-selections he considers unjustified despite meeting published criteria, verbal commitments unfulfilled by selectors, and exclusion from the 4 × 100 relay final at the 2019 World Championships in Doha despite being the fastest French sprinter by record. The objective distance from athletics institutions becomes, for Mouhamadou Fall, the basis of a narrative he now systematically deploys: that of deliberate isolation, orchestrated by federal administrators on account of his “atypical” trajectory and profile.

Restoring this narrative does not mean adopting its terms. An autobiographical discourse selects what it shows and what it omits, and retrospectively confers coherence on a trajectory that did not necessarily possess it (10). The inquiry cannot therefore take up on its own account the argument of distance as Mouhamadou Fall formulates it. Certain material elements of his career are nonetheless attested independently of his testimony. As a sprint specialist (a discipline with limited media visibility in France), Mouhamadou Fall never benefited from stable economic support: “At that point, financially, I was living off the hookah bar,” he told Younes Nezar, referring to his income as a bar manager during his first elite seasons. When recounting his career, Fall consistently emphasizes the precariousness he experienced. This precariousness constitutes the ordinary condition of a large fraction of elite athletes, and is well documented in France (4, 5). What singularizes the case of Mouhamadou Fall is therefore not the precariousness itself, but the use he makes of it today as an argument to legitimize his engagement in the EG. His sponsorship contract with Nike represented his only sustained commercial support [“my sponsor since 2019, who has never let me go, unlike the (French athletics) federation”]. Under these conditions, the distance from institutions widened rapidly. In 2023, Mouhamadou Fall had his licence withdrawn by the federation. Two simultaneous proceedings sealed his rupture with the French sporting system. First, he tested positive for octodrine (a stimulant classified S6 by WADA, of which heptaminol is a metabolite) at the French Championships in Albi in July 2023, a result he attributed to contamination from an American dietary supplement (Raw Nutrition), a pre-workout product he acknowledges not having verified sufficiently despite his obligations. The AFLD Sanction Commission imposed a nine-month suspension, depriving him of the 2024 Paris Olympic Games. Second, three consecutive failures to meet his whereabouts obligations (via the ADAMS software) had already triggered a separate proceeding, taken on appeal by AFLD before the Conseil d’État on May 7, 2024. Fall vigorously contested the official interpretation of these failures, citing a software malfunction, a data-entry error during a training camp in Kenya, and a delayed notification at INSEP, reading in them a deliberate orchestration: “They wanted to bring me down.”

It was in this context that Mouhamadou Fall discovered the Enhanced Games at the end of 2025, when Fred Kerley announced his participation. “I was on Instagram, and when Fred signed, I couldn't believe it until he posted it himself. I sent him a message, what's going on?” He contacted Kerley, gathered information, traveled to Las Vegas to meet EG representatives, and signed quickly. At this point in his career, Mouhamadou Fall is stripped of his licence following two anti-doping rule violations and is unable to compete within the federal framework. It is this situation, which he for his part attributes to a long-standing conflict with the federal apparatus, that makes the EG offer attractive. The language he uses in the promotional video of April 29, 2026, speaks to this sense of recovered freedom: “This was kind of like we’re taking you out of jail and then do what you like to do, run, just run.” The opportunity was also an economic one, the chance to “finally” make a living from his sport. In a direct rejoinder to the federal institutions, on December 15, 2025, Fall posted on Instagram a video of himself getting into a brand new luxury car, captioned: “Revanche Sociale…” [“Social revenge…”, authors' translation]. The phrasing is equivocal.

### 3.2 “Mouhamadou Fall's announced participation throws institutions into disarray”^1^

On October 23, 2025, Mouhamadou Fall announced on social media his intention to participate in the Enhanced Games. The following day, two distinct institutional responses appeared simultaneously, signaling a coordinated reaction. The FFA published a communiqué signed by its president, Jean Gracia, in the register of moral and health risk: Fall's decision was described as being “in total contradiction with the values of sport and athletics,” with the EG denounced for “repudiating integrity, fairness, and the health of athletes”.^2^ The FFA cited its 2025–2028 anti-doping prevention plan and concluded with a declaration of principle: “French Athletics will remain faithful to the Olympic spirit.” The counter-attack was waged entirely on the symbolic terrain: federation leaders used this public statement to prosecute a case of dishonesty against an athlete whose image had already been substantially damaged in recent years.

On the same day, the MSJVA, CNOSF, and AFLD issued a joint declaration whose title was itself revealing: “Enhanced Games: in the event of doping, a French athlete who participates will face sanctions.” This tripartite communiqué recalled that Mouhamadou Fall was “currently suspended by AFLD for two consecutive anti-doping rule violations” and had “held no licence from the French Athletics Federation since 2023”. It explicitly aligned itself with the IOC and WADA athlete commissions' joint statement of June 10, 2025. Substantively, it deployed a rhetoric of civilizational threat: it warned that the twenty-first century could “see the emergence of a new form of institutionalized doping, driven by certain unscrupulous private promoters willing to sacrifice sporting integrity and public health in the staging of an artificial spectacle”. This recourse to dramatization is not specific to this case. Sporting and anti-doping organizations regularly mobilize a crisis register to consolidate their authority and rally public authorities to their cause (11, 12). In response, AFLD announced that it “reserved the right to shortly include Fall in its target group and to conduct the necessary anti-doping tests”. Inclusion in the target group is in itself an ordinary measure for athletes contemplating a return after a sanction. Its mention in a communiqué of this nature nonetheless shifts its scope: stated publicly in this context, it functions less as an administrative procedure than as the announcement of intensified surveillance.

Through these communiqués, French sport leaders issued a blanket rejection of the EG, rendering those who would join it pariahs. This negation of the enterprise's legitimacy enabled them to avoid substantive discussion of the pharmacological or scientific dimensions of the EG's program. No expert report was commissioned, no medical evaluation of the substances involved was undertaken, no attempt was made to distinguish risk profiles according to the products used. The institutional response was essentially performative and juridical: a reaffirmation of rules, accompanied by the threat of controls. This configuration recalls the structural tendency identified by Julie Demeslay (7), who notes that anti-doping governance consistently privileges normative harmonization over individual health protection.

This response was also the one permitted by the state of their means: The IOC and the French federations held very limited leverage over athletes who had chosen to leave their jurisdictions. In the Fall case, the FFA could not deprive him of anything “further,” since he had ceased to be a member in 2023. For its part, AFLD can conduct tests but can only sanction violations of the World Anti-Doping Code on national territory or within regulated competition, and the Enhanced Games operate precisely outside these frameworks. The WADA communiqué of May 22, 2025, had already acknowledged this impasse, calling on governments to “assess whether athletes who admit to taking performance-enhancing substances could be in breach of criminal law or professional rules”. David Douillet, former French Minister of Sports, in declaring that the Enhanced Games “should be banned by law,” provides direct testimony to the absence of institutional purchase over a commercial event that is entirely legal.

Mouhamadou Fall, who maintained that his name had been “dragged through the mud in the traditional system,” intended to use his EG participation as a media platform to rehabilitate his image. His many public statements in the French press afforded significant visibility to arguments that skillfully displaced the debate from doping toward the question of status and remuneration.

### 3.3 Defending, convincing, claiming

Analysis of Mouhamadou Fall's public statements between October 2025 and May 2026 reveals three principles of legitimation.

The first is bodily autonomy. Facing the French sprint community, Fall is careful. He tells Younes Nezar: “Je n’incite personne à se doper, clairement. C’est contre nature, c’est de la triche et c’est immoral. Ce que je fais aujourd’hui, ça ne concerne que moi” [“I’m not encouraging anyone to dope, clearly. It goes against nature, it's cheating and it's immoral. What I’m doing today only concerns me”, authors' translation]. He deliberately maintains ambiguity about his own consumption: “Je ne te dis pas que je vais le faire, et je ne te dis pas que je ne vais pas le faire, là, aujourd’hui” [“I’m not telling you I’m going to do it, and I’m not telling you I’m not going to do it, today”, authors' translation]. This reservation disappears entirely in the EG promotional video released six months later: “I’m going to use the enhancements. I want to see how far I can go, push my body to the limit. I want people to know that this is not something bad. We have professionals, doctors, a lot of people who take care of everything. So yeah, I’m open because I think there's nothing to hide.” The explicit confirmation of substance use, absent from his French-language discourse some months earlier, is stated plainly in this promotional context. This asymmetry is not a contradiction: Mouhamadou Fall adapts his discourse to his audience. Knowing the French public to be attached to norms of “clean” sport, he manages their moral sensibilities accordingly; in the Anglophone promotional register, he fully valorizes his total commitment to the performance enhancement program, framing it as a personal experiment grounded in a fundamental right: the free disposal of one's own body.

The second register concerns health and medical supervision. In his Younivers interview, Fall insists that his use of substances is “monitored by doctors, by scientists,” that it is “not clandestine doping in a garage” but “medicine”.^3^ He articulates this distinction within a broader critique of “regulatory hypocrisy,” invoking caffeine and creatine (once prohibited, now legitimized) to relativize the status of banned substances.^4^ The twelve-week training camp in the United Arab Emirates, documented in a Vanity Fair photoreportage that he shared on Instagram,^5^ and the athletics track in Abu Dhabi on which he trains, materially embody the supervised medicalization he claims, far removed from the clandestine doping denounced by institutions. His Instagram posts extend and render visible what might be termed “selective transparency”: biomechanical analyses with Dr. Setareh, effort tests with a respiratory mask, medical consultations with a visible infusion. On March 20, 2026, he posted his body composition results: 5.9% body fat, 49.6 kg skeletal muscle mass, 91.2 kg total weight. This voluntary biometric transparency is the visual translation of his supervised health discourse: Mouhamadou Fall does not conceal but is not exhaustive either: he documents. In so doing, he turns the institutions' own argument against them: if doping is dangerous because it is clandestine and unsupervised, then what he practices (transparent, documented, supervised) is precisely not doping but performance optimization, which is, moreover, “the very purpose of sport”.

The third register is the value of sporting labor and its remuneration. Speaking to Younes Nezar, Fall develops the following economic calculation: a world champion, in the top 0.01% of the elite, may earn €1,000 a month.^6^ He contrasts this with the conditions offered by the EG (a six-figure signing bonus, a monthly salary, a record bonus) and concludes: “I was made a job offer. I accept it or I don't.”^7^ This formulation repositions sporting performance as ordinary labor subject to contractual logic, breaking with the model of “denied labor” (*travail dénié*) that structures Olympism (13). On December 18, 2025, he posted on Instagram a story reproducing the EG visual (a man facing the figure “$1,000,000”) with a bilingual poll addressed to his audience: “100% HONEST. ACCEPTERAIS-TU/WOULD YOU DO IT?” The question needs no answer: it shifts the moral burden back onto the public.

In the EG promotional video released at the end of April, this hierarchy of motivations is stated explicitly and in a precise order: “The first point was the science. The second was the money, which is a big support, and then you can do your sport the way you really want it. And the third point was I wasn't able to run at this moment.” Far from silencing Mouhamadou Fall, institutional responses unexpectedly afforded him considerable media access. The public condemnation directed at him (resting on the discrediting of his already damaged image) generated press coverage that he turned to his advantage, using it to defend himself and to shift the terms of debate. His private contractual arrangement with the EG, placing him outside international and French sporting jurisdictions, provides a freedom of expression and practice he exercises fully. On March 24, 2026, from Abu Dhabi, he announced on Instagram his ambition to break the European 100-meter record (“European Record pending…”), and the same day shared a carousel citing Max Martin on Fox News: “The world is going to have a much different perspective.” On March 11, 2026, he announced on Instagram having run the 100 m in 9.80 (hand-timed) in training, which would constitute a new European record. Whether or not this performance is ever officially ratified matters little at this stage: it is published, it circulates, and it simultaneously materializes his “social revenge” and a concrete destabilization of the international sporting order.

## 4 Discussion

The Fall case illuminates several dynamics that extend well beyond his individual biography. It brings to light the jurisdictional limits of national sport governance frameworks when confronted with an enterprise operating beyond their reach. Faced with an athlete who is no longer licensed, the French institutions mobilized a register of denunciation whose intensity contrasts with the weakness of the means effectively at their disposal. The Enhanced Games are developed and financially grounded in the United States; their promoters have explicitly invoked the Sherman Antitrust Act to contest attempts by international federations to restrict athlete participation. French and European bodies, whose authority rests on federal affiliation and licence allocation, find themselves in a paradoxical situation: the most credible sanction available to them (competition bans and licence withdrawal) cannot be applied to an athlete who has already exited their purview for several years. The joint declaration of October 24, 2025, acknowledges this implicitly, reducing the threat to the hypothetical prospect of increased anti-doping controls, which could not, in any case, prevent participation in the Enhanced Games themselves.^8^

What was initially framed by European institutions as an ideological fight against doping, through a forceful denunciation of the pharmacological legitimacy of performance enhancement, was progressively displaced. EG leaders and their sporting ambassadors (Mouhamadou Fall for France) undertook to shift it onto the terrain of the recognition of sporting labor, presenting engagement in the EG as a consequence of the insufficient consideration granted to the status and value of that labor in Europe. Our materials show how this subversive narrative circulates, but do not, for now, allow us to establish its success: measuring its reception among the public and among athletes would require a separate inquiry.

The sociology of sport has long documented the tensions between the model of “denied labor” proper to Olympic sport, in which performance is constructed as an expression of passion and vocation rather than work, and the material realities of professional sporting careers (13). By making financial remuneration an explicit and central feature of their offer, the Enhanced Games expose these tensions openly. This enterprise, grounded in capitalist market economics, advances the claim that it alone, through the profits it generates, could remunerate athletes at their “fair value” and thereby protect them from uncertainty, against an Olympic order that would maintain them in permanent financial insecurity. This argument is taken up on their own account by the EG sporting ambassadors. Former 100-meter world champion and now EG employee Fred Kerley explains: “For the first time in my career, I’m receiving all of the resources I need to be successful. For the first time, I’m being treated like the professional athlete that I am.” Mouhamadou Fall defends an identical line. While the FFA invokes “the Olympic spirit” and performance “achieved fairly,” Fall invokes his economic precariousness and repeated exclusions from the French federal system, justifying his EG commitment: “I was made a job offer […] I am finally being offered respect.”

The transatlantic dimension of this cleavage invites consideration of the Enhanced Games not merely as an “atypical” commercial sporting event but as the expression of a specific ideological formation combining libertarian commitments to deregulation and individual sovereignty with transhumanist visions of human enhancement (3, 14). This ideological formation is inseparable from an explicit market strategy: its promoters seek to occupy a central position in the sector of human performance-enhancement products, within a logic in which care no longer aims to treat illness but to optimize the individual body over a life that one seeks to prolong (15). This initiative, more entrepreneurial than sporting, takes effective root in the contemporary political economy of the United States but resonates little with the European tradition of public regulation of sporting bodies and collective governance of health risks. This contrast is concretely illustrated by Mouhamadou Fall himself. In the Anglophone promotional framework, he explicitly confirms his use of performance-enhancing substances and declares “there's nothing to hide”; in the discourse he delivers before the French sprint community, he maintains ambiguity and states he “doesn't encourage anyone to dope”. These arenas produce different discourses because they presuppose and impose different norms.

The results of this article should be understood as preliminary. They open several questions that further investigation will address. The first concerns the “Fall case” as an isolated instance that nonetheless prefigures a broader movement of athletes (at career's end or in situations of economic declassification) for whom the EG offer constitutes a credible exit from the Olympic system. The EG's commercial logic explicitly targets this population, and analysis of the inaugural Las Vegas edition will yield a more complete picture. The second question concerns the recomposition of regulatory authority: will French and European sporting institutions adjust their response to this phenomenon, and if so, how? The third is the question of the legitimate definition of sport itself: by making record-setting an organizing principle and remuneration an explicit right, the Enhanced Games propose a cultural encoding of performance radically different from that patiently constructed by Olympism (9). This alternative encoding of the value of sporting labor (16) warrants sustained attention from the social sciences. The ways in which this model is commercialized by entrepreneurs, embodied by athletes, treated in the media, and received by the public represent so many terrains open to future inquiry.

## Data Availability

The original contributions presented in the study are included in the article/Supplementary Material, further inquiries can be directed to the corresponding author.

## Footnotes

^1^“La participation annoncée de Mouhamadou Fall met les instances dans tous leurs états” [authors’ translation]. *20 Minutes*, October 24, 2025.

^2^“La décision annoncée par Mouhamadou Fall de rejoindre les Enhanced Games est en totale contradiction avec les valeurs du sport et de l’Athlétisme. Cette compétition, qui revendique l’usage de produits dopants, renie l’intégrité, l’équité et la santé des athlètes” [authors’ translation]. FFA communiqué, October 24, 2025.

^3^“Je suis suivi par des médecins, par des scientifiques. Ce n’est pas du dopage clandestin dans un garage. C’est de la médecine” [authors’ translation]. Younivers, October 31, 2025. https://www.youtube.com/watch?v=P9c_DmUhR2g.

^4^“Tous les patients sont dopés, alors n’importe qui est dopé en vrai”/“On soigne des cancers avec la créatine” [authors’ translation]. Ibid.

^5^“Inside the Enhanced Games, Where Athletes Compete on Steroids. And Growth Hormones. And Adderall.” Clara Molot, *Vanity Fair*, April 30, 2026.

^6^“Champion du monde. T’es peut-être 0,01% de l’élite. À l’année, t’es à 1,000 euros [par mois]. Tu te rends compte?” [authors’ translation]. Younivers, November 2, 2025.

^7^“On m’a proposé une offre de travail. Je l’accepte ou je ne l’accepte pas” [authors’ translation]. Ibid.

^8^This impasse is not specific to the French case. The Australian national anti-doping agency, Sport Integrity Australia, adopted an analogous position in a communiqué of May 22, 2025. A systematic comparison of national responses remains to be conducted. https://www.sportintegrity.gov.au/news/media-statements/2025-05/sport-integrity-australia-statement-enhanced-games.

## References

[1] Alexandre O. Tech: When Silicon Valley Remakes the World. Oakland, CA: University of California Press (2025).

[2] Gomez C. Idéologie, autoglorification et brutalité… en toute impunité! Panard. (2026) 9(1):20–31. 10.3917/pan.009.0020

[3] Savulescu J Bostrom N, editors. Human Enhancement. Oxford: Oxford University Press (2009).

[4] Fleuriel S Schotté M. Dépasser l'alternative amateurs/professionnels. Programme pour une histoire sociale des sportifs au travail. Mouv Soc. (2016) 254(1):3–12. 10.3917/lms.254.0003

[5] Fleuriel S Schotté M. Impensables travailleurs sportifs. Pensée. (2020) 401(1):15–23. 10.3917/lp.401.0015

[6] De Léséleuc É Marcellini A. Légitimité vs illégitimité du dopage chez les sportifs de haut niveau. Comment se définissent les limites du non acceptable? Staps. (2005) 70(4):33–47. 10.3917/sta.070.0033

[7] Demeslay J. Harmoniser la lutte antidopage: quelques critiques d'une gouvernance mondiale. Homme Soc. (2016) 199(1):145–58. 10.3917/lhs.199.0145

[8] Ville S. L'argent a tué le sport? Brève analyse historique d'un lieu commun. Savoir/Agir. (2024) 64(1):203–13. 10.3917/sava.064.0205

[9] Ville S. Produire un sport sincère. Genèse d'un idéal de pratique (1870–1925). Genèses. (2023) 131(2):55–80. 10.3917/gen.131.0055

[10] Bourdieu P. L’illusion biographique. Actes Rech Sci Soc. (1986) 62–63:69–72. 10.3406/arss.1986.2317

[11] Zubizarreta Zuzuarregi E Demeslay J. Power relationships between the WADA and NADOs and their effects on anti-doping. Perform Enhanc Health. (2021) 8(4):100181. 10.1016/j.peh.2020.100181

[12] Aust D Krieger J. Communicating in crisis: the International Association of Athletics Federations' public responses to athletics' doping crisis. J Qual Res Sports Stud. (2018) 12(1):263–80.

[13] Fleuriel S. Le travail dénié et les jeux olympiques: entre passions et intérêts. Soc Contemp. (2006) 63(3):85–103. 10.3917/soco.063.0085

[14] Doat D. (2020). Julian Savulescu et la bienfaisance procréatrice. In: Damour F Deprez S Romele A, editors. Le Transhumanisme: Une Anthologie. Paris: Hermann. p. 229–47.

[15] Ménissier T Ohl F Trabal P. Enhanced Games: mais où est vraiment le problème? (2026). The Conversation. Available online at: https://theconversation.com/enhanced-games-mais-ou-est-vraiment-le-probleme-284333 (Accessed June 3, 2026).

[16] Schotté M. La Valeur du Footballeur. Socio-Histoire d'une Production Collective. Paris: CNRS Éditions (2022).

